# Deep amplicon sequencing highlights low intra-host genetic variability of *Echinococcus multilocularis* and high prevalence of the European-type haplotypes in coyotes and red foxes in Alberta, Canada

**DOI:** 10.1371/journal.pntd.0009428

**Published:** 2021-05-26

**Authors:** Maria A. Santa, Andrew M. Rezansoff, Rebecca Chen, John S. Gilleard, Marco Musiani, Kathreen E. Ruckstuhl, Alessandro Massolo

**Affiliations:** 1 Biological Sciences Department, Faculty of Science, University of Calgary, Calgary, Alberta, Canada; 2 Department of Comparative Biology and Experimental Medicine, Host Parasite Interactions (HPI) program, Faculty of Veterinary Medicine, University of Calgary, Calgary, Alberta, Canada; 3 Department of Ecosystem and Public Health, Faculty of Veterinary Medicine, University of Calgary, Calgary, Alberta, Canada; 4 Ethology Unit, Department of Biology, University of Pisa, Pisa, Italy; 5 UMR CNRS 6249 Chrono-environnement, Université Bourgogne Franche-Comté, Besançon, France; Universidad de la República Uruguay: Universidad de la Republica Uruguay, URUGUAY

## Abstract

*Echinococcus multilocularis* (*Em*) is a zoonotic parasite considered a global emergent pathogen. Recent findings indicate that the parasite is expanding its range in North America and that European-type haplotypes are circulating in western Canada. However, genetic analyses are usually conducted only on a few parasites out of thousands of individuals within each definitive host, likely underestimating the prevalence of less common haplotypes. Moreover, mixed infections with several mtDNA haplotypes in the same host have been reported, but their relative abundance within the host was never estimated. We aimed to 1) estimate the frequency of co-infections of different *Em* haplotypes in coyotes (*Canis latrans*) and red foxes (*Vulpes vulpes*) from western Canada and their relative abundance within the definitive hosts, 2) detect less prevalent haplotypes by sampling a larger proportion of the parasite subpopulation per host, and 3) investigate differences in the distribution of *Em* haplotypes in these main definitive hosts; foxes and coyotes. We extracted DNA from ~10% of the worm subpopulation per host (20 foxes and 47 coyotes) and used deep amplicon sequencing (NGS technology) on four loci, targeting the most polymorphic regions from the mitochondrial genes *cox1* (814 bp), *nad1* (344 bp), and *cob* (387 bp). We detected the presence of mixed infections with multiple *Em* haplotypes and with different *Echinococcus* species including *Em* and *E*. *granulosus s*.*l*. genotypes G8/G10, low intraspecific diversity of *Em*, and a higher abundance of the European-type haplotypes in both hosts. Our results suggest a population expansion of the European over the North American strain in Alberta and a limited distribution of some European-type haplotypes. Our findings indicate that deep amplicon sequencing represents a valuable tool to characterize *Em* in multiple hosts, to assess the current distribution and possible origins of the European strain in North America. The potential use of next-generation sequencing technologies is particularly important to understand the patterns of geographic expansion of this parasite.

## Author summary

*Echinococcus multilocularis* is a tiny tapeworm whose adults live in the intestines of wild and domestic canids and shed their eggs with the host feces. If accidentally ingested by people, eggs can cause human alveolar echinococcosis (AE), a highly lethal parasitic disease. Despite *Em* being common in North America, only two locally acquired AE human cases were reported in North America until 2013. However, in the last 6 years, 14 new cases have been detected in Alberta, Canada, and recent findings showed that the European strain of this parasite is now common in wildlife in this province, which could potentially increase the risk of infection in humans and domestic animals. In this study, we genetically characterized *Em* in coyotes and red foxes to assess the extent of the European-type strain distribution in three Canadian provinces. We used deep amplicon sequencing, an innovative approach that allows us to detect co-infections and the presence of less prevalent genetic variants by sampling a larger proportion of the parasite subpopulation per host. Our study showed an overwhelming prevalence of the European strain over the native North American strain in both hosts, and a limited geographical distribution of some European genetic variants, likely due to multiple introduction events of this invasive strain.

## Introduction

Echinococcosis is a chronic zoonotic disease, caused by the larvae of *Echinococcus* spp. that affects humans as well as domestic and wild animals, representing a significant public health concern in many countries throughout the world [1,2]. Due to their worldwide distribution [3], the genetic characterization of these parasites is key to assess public and animal health risk, as individual genetic variants may have different pathogenic effects in their hosts, including humans [4].

In recent years, whole-genome sequencing of mitochondrial (mtDNA) and nuclear DNA (nDNA) has been completed for several *Echinococcus* genotypes, providing new insights into the biology, development, and phylogeny of this taxonomic group [5–7]. However, the study of *Echinococcus multilocularis*, the causative agent of alveolar echinococcosis (AE), has been slow due to the use of low throughput and time consuming diagnostic methods [8]. Additionally, it has been difficult to find highly discriminatory genetic markers to study the parasite’s population genetics and the ecological and evolutionary process, underlying changes in the geographic distribution and incidence of this parasite [9].

The intra-specific genetic diversity of *E*. *multilocularis* has been described using multiple mtDNA and nDNA markers, which typically exhibited low polymorphism compared with other *Echinococcus* species [10,11]. Based on three mtDNA genes (*cob*, *nad2*, *cox1*), four well-differentiated genetic strains were described worldwide (Asian, European, North American and Mongolian) by Nakao et al.[11]. In that study, only two haplotypes of the North American strain (N1 and N2) were found respectively in the two historical endemic regions: in the Northern Tundra Zone in Alaska and the Canadian Arctic; and in the Northern Central region, which includes the southern area of three Canadian provinces (Alberta, Saskatchewan and Manitoba), and 13 contiguous states in the northern USA [11]. However, only 17 samples from 13 hosts from North America were included in that study, with two samples from Alaska corresponding to the Asian strain.

Recent studies in Canada have revealed the circulation of European-type haplotypes in wild canids in Saskatchewan (SK) and Alberta (AB), as well as in previously non-endemic areas of British Columbia (BC), suggesting an introduction of this strain, and a general expansion of the parasite’s distribution in western Canada, but also in eastern areas like Ontario [12–14]. Besides, there has been an alarming increase in human AE cases in the province of Alberta since 2013, with an estimated incidence of 0.059 cases/100,000 inhabitants/year, which is comparable to those found in endemic areas of Europe [13,15,16]. Furthermore, the European-type haplotype ECA, with one single nucleotide polymorphism in *cob* gene, unique to Canada, was identified as the causative agent of human AE cases, suggesting local acquisition of these infections and the emergence of a new endemism [13]. Moreover, it has been presumed that the North American strain could be less virulent and/or pathogenic than the European variants, due to the historically lower number of human cases in North America compared to Europe [11]. Therefore, it is highly relevant to genetically characterize *E*. *multilocularis* populations in multiple hosts throughout western Canada, to assess the current distribution of the European strain.

One methodological limitation in the study of these parasites is the sampling adequacy problem in characterizing the *Echinococcus* species and strains distribution across space and hosts. Genetic analyses of *E*. *multilocularis* are usually conducted on only a few parasites from each definitive host, whereas the worm burden per definitive host can go as high as hundreds of thousands of worms [17,18]. Therefore, this could lead to an underestimation of the genetic diversity and prevalence of less common genetic variants [19]. Indeed, mixed infections with several genetic profiles in the same host have been commonly reported in red foxes from Europe and Asia, using the microsatellite EmsB as a genetic marker [19–21]. Moreover, Knapp et al. [22] observed that a high worm burden might be indicative of multiple infections, and the more worms were analyzed from one fox, the more genetic profiles were detected, thus, supporting the hypothesis of an inadequate estimation of within-host diversity.

In recent years, the increased use of next-generation sequencing (NGS) technologies for the study of helminth parasites has helped to overcome limitations in their identification and surveillance, allowing the quantification of species diversity more efficiently and accurately, thus exploring in greater depth the parasite communities in multiple hosts [e.g. 23]. In particular, deep amplicon sequencing has been implemented in large scale surveys of nematode communities in domestic and wild ruminants [24,25], showing a higher sensitivity to detect species with low prevalence, and providing accurate estimations of their relative abundance.

In this study, we developed a deep amplicon sequencing assay to 1) estimate the frequency of co-infections of different *Em* haplotypes in western Canada and their relative abundance within hosts, 2) detect less prevalent haplotypes by sampling a larger proportion of the parasite subpopulation per host, and 3) investigate differences in the prevalence and intensity of infection of *Em* haplotypes between the foxes and coyotes. Ultimately, we aimed to highlight the advantages and potential use of NGS to study the genetic diversity and distribution of *E*. *multilocularis* and in general of *Echinococcus* spp. in large-scale studies.

Using this approach, we could estimate the relative abundance of each haplotype in the two main species of definitive hosts and detect low prevalent haplotypes. We found a lower intraspecific diversity of *E*. *multilocularis* than previously reported in western Canada, a higher abundance of the European-type haplotypes, and a higher frequency of *Em* co-infections.

## Materials and methods

### Parasite material

**Fig 1:**
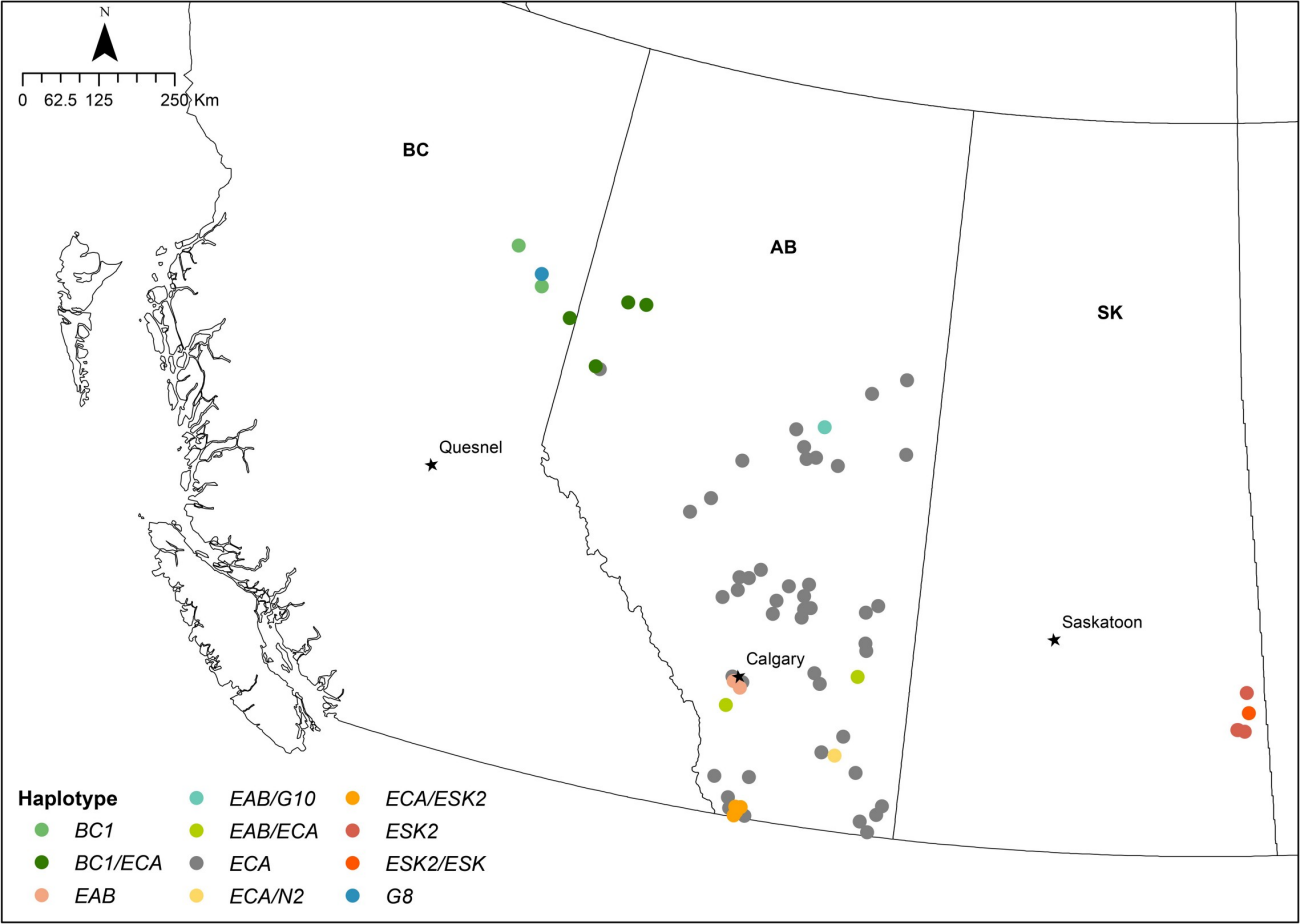
Distribution of *E*. *multilocularis* and *E*. *granulosus s*.*l*. haplotypes in western Canada. The map shows the distribution of *E*. *multilocularis* and *E*. *granulosus s*.*l*. haplotypes in the provinces of British Columbia (BC), Alberta (AB), and Saskatchewan (SK). Different colours are used to represent each haplotype in single and mixed infections. The *Em* EAB and N2 were only found in Alberta, and the ECA was the most prevalent in that province. Haplotypes from British Columbia (BC1) were not found in Saskatchewan and the ESK2, the most prevalent haplotype in Saskatchewan, was not found in BC. Basemap layer available from: https://www12.statcan.gc.ca/census-recensement/2011/geo/bound-limit/bound-limit-2016-eng.cfm.fig.

We used *Echinococcus* spp. specimens collected from intestinal tracts of coyote and red fox carcasses of either road-killed or animals trapped for other purposes, collected between 2012 and 2017, from rural and urban areas in Western Canada (Fig 1). Trapped animals were collected from licensed trappers, with the collaboration of the Alberta Trappers Association. Intestinal tracts were previously screened for the presence of *Echinococcus* spp. worms using the scraping, filtration, and counting technique (SFCT) [17], with some modifications [26]. *Echinococcus* worms were collected through sieves (250μm and 75 μm) and preserved in a final volume of 50 ml of 70% ethanol (mixed worm samples). In total, we included in this study mixed worm samples from 47 coyotes and 20 foxes: 38 coyotes and 20 foxes from Alberta, 5 coyotes from Saskatchewan and 4 from British Columbia, with a worm burden between 500 and 230,000 in coyotes, and 50 and 20,000 in red foxes.

### DNA extraction

Genomic DNA lysates were prepared from each mixed worm sample following a protocol previously described [26]. Total DNA extraction was performed on aliquots of approximately 10% of the worm subpopulation per host, by processing 5ml of each 50ml mixed worm sample. By extracting DNA from the same percentage of worms, we aimed to maximize the number of worms sequenced per host. At the same time, we wanted to avoid the time-consuming process of re-counting individual worms, since the small size of the worms and the presence of intestinal detritus in the filtrate difficult their identification. The E.Z.N.A stool DNA kit (Omega bio-tek, US) was used for the DNA extraction to remove potential PCR inhibitors, commonly found in intestinal content samples, following the manufacturer’s recommended protocol, after centrifugation and evaporation of ethanol from the samples. The remaining pellet without ethanol was weighted and portioned to process 200 mg per extraction. If needed, more than one extraction was performed per sample, according to the amount of pellet, and then combined in only one DNA aliquot. The concentration of DNA lysates was measured with NanoPhotometer NP80 (Implen, USA) and samples were diluted to ~10 ng/μl before addition to PCR reactions.

### Primer design

Multiple forward and reverse oligonucleotide primers were designed to PCR-amplify specific loci from the *cob*, *nad1* and *cox1* mitochondrial genes of *E*. *multilocularis*, using the Primer3 software (http://primer3.ut.ee/). Primers previously reported by Nakao et al. [11] and Trachsel et al. [27] were also included. We selected four highly polymorphic regions (344 to 409 nucleotide sites) to be able to identify multiple haplotypes of *E*. *multilocularis*. The location of primers and target regions are shown in S1 Appendix. Illumina adapters were added to the selected primers, obtaining four forward and four reverse primers per locus with 0–3 N’s included between the adapter and the primer-binding region [24]. The primer design was as follows: 5’-Illumina adapter sequence– 0-3N’s–Primer binding regions -3’. The forward Illumina adapter sequence used was: 5’-TCGTCGGCAGCGTCAGATGTGTATAAGAGACAG-3’, and the reverse Illumina adapter sequence was: 5’-GTCTCGTGGGCTCGGAGATGTGTATAAGAGACAG-3’. The Illumina adapter oligonucleotide sequences were obtained from the Illumina Adapter Sequences document (February 2016; Oligonucleotide sequences 2016 Illumina, Inc.). PCR primers are summarized in S1 Table.

### Deep amplicon sequencing

Forward and reverse adaptor primers per locus were pooled together in equal proportions to obtain a final concentration of 10 μM, and used for PCR under the following conditions: 5 μL NEB Q5 High-Fidelity DNA Buffer (5X) (New England Biolab, Ipswich, MA, USA), 1.25 μL or each forward and reverse primers (10 μM), 0.5 μL dNTPs (10 mM), 0.25 μL Q5 High-Fidelity DNA Polymerase (0.7 U), 14.7 μL ddH2O, 2 μL of worm lysate. The thermocycling parameters were 98°C for 3 minutes, followed by 32 cycles of 98°C for 8 seconds, locus specific annealing °C for 20s, 72°C for 20s, followed by a final extension of 72°C for 2 min. Annealing temperatures are summarized in S1 Table.

PCR reactions were run separately for each locus, and each sample was run in duplicate, including two negatives controls in each run. PCR products were purified with AMPure XP Magnetic Beads (1X) (Beckman Coulter, Inc.), following the manufacturer’s recommended protocol. Illumina indices and P5/P7 sequencing tags (Oligonucleotide sequences 2016 Illumina, Inc.) were then added to the PCR amplicons. The P5/P7 tags were combined to make up unique forward/reverse barcode combinations to identify individual samples. The following PCR conditions were used: 5 μL of KAPA HiFi HotStart Fidelity Buffer (5x) (KAPA Biosystems, USA), 1.25 μL of Forward Primer (S501-S520) (10 μM), 1.25 μL of Reverse Primer (N701-N726) (10 μM), 0.75 μL of dNTPs (10 mM), 0.5 μL KAPA HiFi HotStart Polymerase (0.5 U), 13.25 μL of ddH2O, and 3 μL of first-round clean PCR product as template. The thermocycling parameters were: 98°C for 45s, followed by nine cycles of 98°C for 20s, 63°C for 20s, 72° C for 2 min. This was followed by a second round of bead purifications of indexed amplicons.

The concentration of each sample was measured using the NanoPhotometer NP80 (Implen, USA). Then, a master sequencing mix was created by pooling 20 ng of purified PCR product from each sample, therefore obtaining a pooled library. The final concentration of the library was assessed with the KAPA qPCR Library Quantification Kit (KAPA Biosystems, USA), following the manufacturer’s recommended protocol. The library was then diluted to 4 nM based upon the KAPA qPCR results. The final pooled library was run on an Illumina MiSeq Desktop Sequencer using a 600-cycle paired-end reagent kit (MiSeq Reagent Kit, v3, MS-102-3003) at a concentration of 15 pM, with the addition of 20% PhiX control v3 (Illumina, FC-110-3001).

### Bioinformatic analysis

Sequence data were analyzed using the open-source R package Dada2, pipeline version 1.8 [28] and the application Cutadapt 2.10 [29]. Samples were automatically demultiplexed with the MiSeq, based on the supplied index combinations. Cutadapt was used on the raw paired-end reads to detect and trim off regions matching the primer sequences, discarding unmatched reads. Trimmed reads were analyzed following the Dada2 pipeline workflow, which included filtering, dereplication of identical reads, sample inference, chimera identification, merging of paired-end reads and construction of an amplicon sequence variant (ASV) table. During filtering, a maximum of 2 and 5 expected errors, predicted by the quality scores of sequences, were allowed for the forward and reverse reads, respectively, discarding reads shorter than 200 bp according to the length of the amplicon expected [28]. Detailed information regarding the analysis pipeline can be found at www.nemabiome.ca.

For haplotype assignment, ASVs sequences were aligned in Geneious 10.0.9 (Biomatters Ltd, New Zealand) and compared with *Echinococcus* spp. reference sequences available in the GenBank database, using the Nucleotide BLAST tool (https://blast.ncbi.nlm.nih.gov/Blast.cgi). The proportion of ASVs per sample at each locus was calculated by dividing the number of raw reads per ASV by the total number of reads per sample. ASVs with a proportion of < 0.05% per sample were considered as trace amplicon contamination [24]. Results of each locus were concatenated to identify the final haplotype, and their final relative proportion in each sample corresponds to the mean of the proportion at all loci. The identity of the concatenated sequences was confirmed by Sanger-sequencing the full length of *cob*, *cox1*, and *nad2* genes of individual worms from the same hosts, using primers and PCR conditions reported by Nakao et al. [11] (S2 Table). In cases of haplotypes with low relative abundance within a host, we confirmed the haplotype’s identity by comparing it with other samples in which the respective haplotype was identified from individual worms. Therefore, all new haplotypes found using deep sequencing were confirmed by Sanger sequencing.

### Statistical analysis: Foxes *vs*. coyotes

We compared the frequency of *Em* co-infections and single infections between coyotes and foxes from Alberta using Fisher’s exact test. In cases of co-infections, the relative abundance (percentage of reads) of the most prevalent haplotypes (n ≥ 3) was compared between both hosts, using the Mann-Whitney U test. The same test was used to compare the differences in intensity of infection (worm burden) between coyotes and foxes in cases of single infections and co-infections. The alpha-diversity of *E*. *multilocularis* in Alberta, comparing coyotes and foxes, was calculated using a rarefaction analysis to calculate the inverse Simpson index based on the lowest number of reads per host (8000 reads) [30]. The beta-diversity between hosts was calculated using the Bray-Curtis dissimilarity index [31]. Statistics were performed in SPSS Statistics (IBM Corp. IBM SPSS Statistics for Windows, Version 26.0. Armonk, NY: IBM Corp). Diversity indices were calculated using the R statistical software (R Development Core Team, 2020) and the package *vegan* [32].

## Results

### Deep sequencing analysis

**Fig 2:**
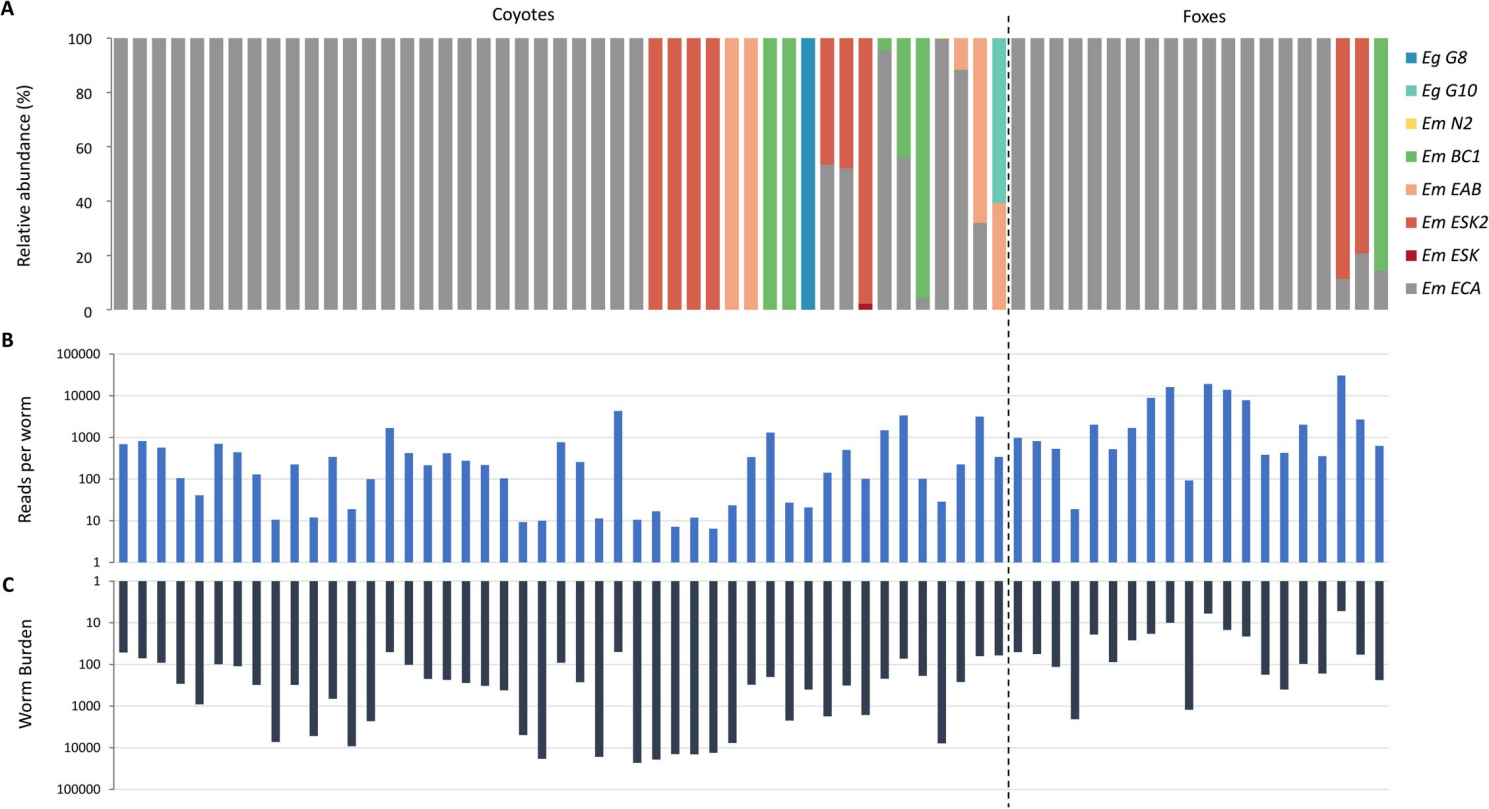
Deep amplicon sequencing assay results. **Panel A.** Relative abundance (%) of *E*. *multilocularis* and *E*. *granulosus s*.*l*. haplotypes per host. The percentage of reads corresponding to different ASVs (amplicon sequence variant) at each locus (*cob*, *cox1_A*, *cox1_B*, *nad1*) was concatenated to identify the final haplotype and their final relative proportion in each sample. We identified in total eight haplotypes: *E*. *multilocularis (Em)* ECA, ESK, ESK2, EAB, BC1, N2, and the *E*. *granulosus s*.*l*. *(Eg)* G8 and G10. The ESK2 was the only new *Em* haplotype. **Panel B and C**. Comparison of the total reads per worm (**A**) and the number of worms per sample (**B**). According to the number of worms processed per host, we obtain a read depth of approximately 7 to more than 30,000 reads per worm. The average read depth per sample was 117,479 (range 8,351–325,480).

Between 8,351 and 325,480 (*M* = 117,479; *SD* = 79,745) sequence reads were generated per sample, representing between 7 and 30,710 reads per worm, depending on the total worm burden per host (Fig 2). Five ASVs were identified per locus: *cob* (387 bp), *cox1_A* (405 bp), *cox1_B* (409 bp), which mapped to *E*. *multilocularis* (European and North America strains), and *E*. *granulosus s*.*l*. (*Eg*) genotypes G8/G10. The percentage identity per locus was 100% for *Em* sequences, and between 99.2% to 99.7% for *Eg*. Only one ASV was identified at the locus *nad1* (344 bp), corresponding to the *Em* haplotype "E". This haplotype was previously found by Gesy et al. [33] in most Canadian regions investigated, included in worms from British Columbia identified as European-type strains based also on *cox1*, *cob* and *nad2* markers. After concatenating the results of the four loci per sample (in total 1201 bp), we identified eight haplotypes: five *Em* European-type (ECA, ESK, ESK2, EAB, BC1), one *Em* North American strain (N2) (Table 1), and *E*g G8 and G10. The ESK2 was the only new *Em* haplotype, with only two different SNPs in *cob* and *cox1* compared to the E4 European haplotype (AB461395.1), whereas the other haplotypes were reported previously [11,13,34].

**Table 1:** Single nucleotide substitutions of *cob*, *nad1* and *cox1* genes found in six haplotypes of *Echinococcus multilocularis*.

	*cob*						*nad1*			*cox1*									
Haplotype	72	81	165	235	369	380	231	365	490	547	585	675	688	735	760	800	822	982	1122
NC_000928.2*	T	A	T	A	T	G	C	C	A	A	G	T	C	T	A	C	G	C	G
*Em N2*	C	.	.	.	.	A	T	T	G	.	A	.	T	G	G	T	.	.	.
*Em BC1*	.	.	C	.	C	A	T	.	.	.	.	G	T	.	.	.	A	T	.
*Em EAB*	.	G	C	G	C	A	T	.	.	.	.	G	T	.	.	.	A	.	A
*Em ESK*	.	G	C	G	C	A	T	.	.	G	.	G	T	.	.	.	A	.	.
*Em ESK2*	.	.	C	.	C	A	T	.	.	G	.	G	T	.	.	.	A	.	.
*Em ECA*	.	G	C	G	C	A	T	.	.	.	.	G	T	.	.	.	A	.	.

The substitutional sites are numbered from the initiation codon of each gene. The total length of the concatenated sequences was 1201 bp: *cob* (387 bp), *nad1* (344 bp), *cox1_A* (405 bp), and *cox1_B* (409 bp).

* A complete gene sequence from Asia, accession number NC_000928, was used as the reference sequence.

The distribution of all the haplotypes found in western Canada is shown in Fig 1. The *Em* ECA was found widely distributed throughout Alberta. The *Em* BC1 was found in British Columbia and the border with Alberta, in the northwestern area. The *Em* ESK2 is distributed in Saskatchewan and southern Alberta, along with the *Em* ESK. The *Em* EAB and *Em* N2 were only found in Alberta. The *Em* ECA haplotype was the most frequent in Alberta, with a prevalence of 92.1% (95% CI 78.6–98.3) in coyotes, and 100% (95% CI 83.2–100) in foxes. In coyotes from British Columbia, the *Em* BC1 was the most frequent, with a prevalence of 75% (3/4), and in coyotes from Saskatchewan, the most prevalent was *Em* ESK2 founded in 100% of samples (5/5). The prevalence and relative abundance of each haplotype are summarized in Table 2. New generated sequences have been submitted to GenBank (Accession numbers: MW591188-MW591189, MW602654-MW602657).

**Table 2:** Prevalence and mean relative abundance of *E*. *multilocularis* and *E*. *granulosus s*.*l*. haplotypes found in western Canada.

Host	Location	n	Haplotype	No. positive	Prevalence % (95% CI)	Mean relative abundance in co-infections (SD)
Coyotes	Alberta	38	*Em ECA*	35	92.1 (78.6–98.3)	68.2 (26.1)*
			*Em EAB*	5	13.2 (4.4–28)	39.7 (28.1)
			*Em ESK2*	2	5.3 (0.6–17.7)	47.3 (0.9)
			*Em BC1*	1	2.6 (0.07–13.8)	24.3 (7.9)
			*Em N2*	1	2.6 (0.07–13.8)	0.2 (N/A)
			*Eg G10*	1	2.6 (0.07–13.8)	60.6 (N/A)
	British Columbia	4	*Em BC1*	3	75 (19.4–99.4)	95.8 (N/A)
			*Em ECA*	1	25 (0.06–80.6)	4.3 (N/A)
			*Eg G8*	1	25 (0.06–80.6)	N/A
	Saskatchewan	5	*Em ESK2*	5	100 (47.8–100)	97.8 (N/A)
			*Em ESK*	1	20 (0.05–71.6)	2.2 (N/A)
Red foxes	Alberta	20	*Em ECA*	20	100 (83.2–100)	15.5 (4.8)
			*Em ESK2*	2	10 (1.2–31.7)	83.9 (6.7)
			*Em BC1*	1	5 (0.01–24.9)	85.6 (N/A)

Significant difference in mean relative abundance in co-infections between coyotes and foxes from Alberta are indicated by * (*p <*0.05, Mann-Whitney U test)

### Co-infections and distribution of haplotypes in foxes and coyotes

The overall frequency of co-infections was 15% (3/20) for foxes and 21% (10/47) for coyotes, with the most frequent combination between *Em* ECA/ESK2 and *Em* ECA/BC1 (31% each one), and only one co-infection with *Em* (EAB) and *Eg* (G10) (Table 2 and Fig 2). The North American haplotype *Em* N2 was only found in one sample and had the lowest relative abundance (0.2%). The difference in the frequency of *Em* co-infections comparing coyotes [18% (7/38)] and foxes [15% (3/20)] from Alberta, was not statistically significant (Exact sig., 2-sided, *p* = 1). However, the relative abundance of *Em* ECA (the most prevalent haplotype), in cases of co-infection, was statistically significantly higher in coyotes (*n* = 7, *M* = 68.2, *SD* = 26.1), than foxes (*n* = 3, *M* = 15.5, *SD* = 4.8; *U* = 0, *p* = 0.017). Likewise, in single infections with the ECA haplotype, the difference in intensity of infection (worm burden) between hosts, was statistically significantly higher in coyotes [*n* = 28, *Mdn* = 2989 (41,621)], than foxes [*n* = 17, *Mdn* = 560 (1,510); *U* = 98.5, *p* = 0.001]. The difference in the worm burden in co-infections compared to single infections was not statistically significant for coyotes (*p* = 0.19) and foxes (*p* = 0.47). Comparing the distribution of *Em* haplotype within the two hosts, the haplotype diversity was similar, based on Bray-Curtis dissimilarity (0.35), and the alpha inverse Simpson index showed low values of *Em* diversity in coyotes (1.32) and foxes (1.30) from Alberta.

## Discussion

In our study, we were able to detect and estimate the frequency of mixed infections with multiple *E*. *multilocularis* haplotypes, as well as to detect co-infections with other *Echinococcus* species. Using deep amplicon sequencing, an innovative approach to study the genetic diversity of the worm subpopulation within each host, we could estimate the relative abundance of each haplotype in the two main species of definitive hosts and detect low prevalent haplotypes. We found a lower intraspecific diversity of *E*. *multilocularis* than previously reported in western Canada [12,33], a higher abundance of the European-type haplotypes in the two hosts foxes and coyotes, and a higher frequency of *Em* co-infections. Particularly, we confirmed the high prevalence of the ECA haplotype in Alberta, a region that is facing an unprecedented emergence of alveolar echinococcosis in humans [13]. Our results suggest a population expansion of the European over the North American strain in that province and a possible host partitioning by this invasive strain.

In Canada, the genetic diversity and distribution of *E*. *multilocularis* have been typically explored using classic mtDNA markers based on PCR cloning and Sanger sequencing. Compared to these techniques, the use of deep amplicon sequencing on a higher number of individual worms per host, from multiple within-host parasite sub-populations, resulted in more robust results, avoiding errors due to low sample input and amplification bias. This way, by having a higher depth of reads per base pair (mean of 117,479 reads per sample), we likely avoided an underestimation of haplotypes with low relative abundance, providing at the same time more accurate and reliable quantification of *E*. *multilocularis* diversity. Additionally, the technical validity of results was confirmed by running each sample in duplicate and avoiding cross-contamination by running PCR reactions separate for each locus.

In a previous study, upon sequencing a region of the *nad1* gene from parasites collected in wild intermediate and definitive hosts in northern and western Canada, Gesy et al. [33] found 17 new haplotypes, representing a higher diversity than expected, as only two haplotypes had been reported before using that locus. Interestingly, our findings showed no variation in the same gene from all samples collected in western Canada. Our sequences matched the haplotype "E", which was previously identified by Gesy et al. [33] in intermediate and definitive wild hosts in the provinces of Saskatchewan, British Columbia, Alberta, Manitoba and Nunavut, being this, the second most prevalent haplotype found in that study. Moreover, this haplotype also matched previously published sequences of worms identified as European-type in a dog from BC (JN371771). When comparing the number of *Em* haplotypes found in previous studies in Saskatchewan, with our results in Alberta (the most extensive area investigated), using *cox1*, *nad2* and *cob* markers, the total number of haplotypes found in Saskatchewan was higher (7 North American, 1 European-type), with the North American strain being more prevalent [56% (23/41)] than the European [12,33]. Surprisingly, we only found one coyote co-infected with the North American strain (N2) in Alberta, with a relative abundance of only 0.2%, compared to the overwhelming prevalence of European-type haplotypes, with the most dominant being the ECA, which has been associated with the most recent human cases of AE in the province of Alberta [13]. The observed differences may suggest some degree of competitive interactions between the European and North American strains in Alberta, as an alternative to a stochastic process. These competitive interactions can be mediated by factors such as the abundance of host resources (exploitation competition), host immune responses (apparent competition), and specific parasite strategies for survival, growth and reproduction [35]. Since it has been presumed that the North American strain is less virulent than the European variants [11], the establishment of the European strain in western Canada into naïve host species, such as coyotes, may have had affected the transmission of local parasite strains, through direct and indirect effects on interactions within these predator-prey host communities [36].

The higher prevalence of North American haplotypes described before, compared with our findings, could also be related to the extension of geographical sampling area in each province, the sequencing methodology used, and possibly to different routes of invasion of the European strain. The limited distribution of some haplotypes, like EAB that was only found in Alberta, and strains from British Columbia not being present in Saskatchewan and vice versa, could be an indication of multiple introduction events of the European strain and some degree of isolation by distance, facilitated through translocation of domestic dogs from other endemic areas [34], and/or introduced red foxes imported for sport hunting [37], although more extensive sampling in British Columbia and Saskatchewan, and the use of multiple genetic markers are necessary to better understand the spatial distribution patterns of these strains.

Several studies have demonstrated co-infections of multiple *Echinococcus* genotypes in individual hosts such as wolves (*Canis lupus*) [38], foxes [21] and coyotes [26]. Using the microsatellite EmsB as genetic marker, different genetic profiles of *E*. *multilocularis* worms were found for example, in 52% of 25 foxes in France [22], and 17.6% of 91 foxes in Poland [19]. This phenomenon has been commonly associated with intense predation activity of definitive hosts leading to an increased likelihood of ingestions of multiple infected intermediate hosts (e.g. small rodents), or the presence of protoscoleces belonging to several genetic variants in an intermediate host [20,21]. Likewise, the definitive host species and their prey preferences, the size of the geographic area investigated, and the type of genetic marker used to discriminate genetic variants should be considered. The multilocus microsatellite EmsB is generally a more sensitive tool with a higher discriminatory power than classical nuclear and mitochondrial targets, detecting a higher genetic variability than mtDNA markers at continental and at a local scale [9], hence potentially increasing the likelihood of detection of multiple genetic profiles in the same host. For example, in previous studies in Canada using mtDNA markers, co-infections of multiple *E*. *multilocularis* haplotypes were reported only in artic foxes (*Vulpes lagopus*) with a frequency of 10.7% (3/28) [33]. In our study we found a frequency of *Em* co-infections in foxes (15%) and coyotes (18%) from Alberta similar to the frequency found in red foxes in Poland (17.6%, based on EmsB), an area previously not recognized as endemic, with lower *E*. *multilocularis* diversity compared to European historical endemic regions [19].

Regarding mixed infections with multiple *Echinococcus* species, we detected only one co-infection with *E*. *multilocularis* and *E*. *granulosus s*.*l*. G10 in a coyote, which could be related to a lower amplification efficiency due to the use of non-species specific primers for *Eg*. As reported by Avramenko et al. [24], differences in amplification efficiency due to sequence variation might be expected, whereby correction factors based on mock parasite communities could be calculated to avoid species-specific biases. Despite this, we did not calculate correction factors for each species in the present study since we were focused on the detection of *E*. *multilocularis* haplotypes. In our case, the depth of sequence data per sample, the use of replicates and consistent results between assays, helped to minimize a representation bias of different haplotypes. However, we recommend the use of species-specific primers to detect different *Echinococcus* species, depending on the purpose of the study. Moreover, the presence of worms in different stages of development in cases of co-infection might alter to some extent the relative abundance estimates of each haplotype, since gravid worms would have more genome copies compared to non-gravid. For future analyses, it would be valuable to compare different approaches using gravid worms *vs*. non- gravid to see their impact on each haplotype’s relative abundance in the same host.

When comparing the frequency of *Em* co-infections in foxes and coyotes from Alberta, there was no significant difference, possibly due to their similar rodent-based diets, or because of the relatively small sample size of foxes. Likewise, there was no significant difference in the number of worms comparing single and mixed infections in foxes and coyotes. This could be related to multiple infections by the same haplotypes, which might explain a very high worm burden even if only one haplotype was found. While red foxes are typically generalists and opportunistic predators, in some areas, their diet can consist mainly of small rodents [39], thus increasing the chances of re-infection by two or more parasites with different genetic variants. Likewise, although coyotes may also prey upon larger animals, depending on prey abundance and seasonality [40,41], a study showed that small mammals constituted the bulk diet for urban coyotes in Calgary (AB) during most seasons. Most of these prey species were competent hosts for *E*. *multilocularis*, resulting in a mean of 101.7 intermediate hosts ingested by a coyote per season [41].

When comparing the richness and evenness of *Em* haplotypes within the two hosts in Alberta, the diversity was low for both, being the ECA the most prevalent haplotype in the two hosts. However, the intensity of infection of the ECA haplotype in mixed and single infections, was significantly higher in coyotes compared to foxes, which could be related to biological and ecological factors, such as physiology, immunology and behaviour, influencing species-specific interactions in this multi host-parasite system [42,43]. Nevertheless, differences in virulence, infectivity, and specificity between different strains, as well as the role of different hosts in the transmission of the parasite, have not yet been explored in depth. In recent years, genome-wide transcriptome studies using NGS have been conducted trying to understand the mechanisms that regulate the parasite development and their role in host-parasite interactions [44,45]. Despite these advances, there is still a lack of robust methods for conducting functional genomic analyses to annotate all the parasite genes, and thus investigate the potential differential effects of each strain in multiple hosts.

Recently, Laurimäe et al. [7] proposed the analysis of complete mitochondrial genomes as an option to obtain deeper insights into the genetic diversity, phylogeny and population structure of *Echinococcus granulosus s*.*l*., finding significantly better phylogenetic and geographic resolution (e.g. higher diversity and haplogroups) than using short mtDNA sequences. However, whole-mitogenome sequencing can be rather expensive and time-consuming. Hence, multiple highly diverse short sequences can still be useful for simple haplotype assignments, particularly when wanting to generate high sequencing depth from large numbers of samples. As demonstrated with our study, the use of deep amplicon sequencing using multiple mtDNA markers on the parasite subpopulation per host, allows us to estimate the frequency of mixed infections and to detect haplotypes with a low relative abundance within each host. Pooling samples helps to maximize the number of individual worms sequenced from multiple host-populations, optimizing cost and increasing analytical power and resolution. This could be of particular importance in the study of the patterns of geographic expansion of these parasites, and the role of each host in their transmission. In the last decade, the rapid development of NGS technologies has opened up new opportunities for a variety of genomic investigations into parasites of public health interest, becoming routine in biological research. The latest advances in this technology allow sequencing of long reads from single DNA molecules, using minimal amounts of DNA, reducing costs and optimizing throughput time. Since this parasite continues to be a significant threat to human and animal health, the use of these technologies will help to advance our understanding of the epidemiology of *E*. *multilocularis*, thus helping to improve the surveillance, prevention, and control of this parasite.

## Supporting information

S1 Appendix Multiple sequence alignment of *cob*, *cox1* and *nad1* genes. We aligned previously reported sequences found in North America of *cox1*, *nad1* and *cob* genes to identify highly polymorphic regions and select the primers to amplify the target regions. The primers chosen per locus are shown underlined. The arrows indicate the 5’ to the 3’ direction.
(DOCX)

S1 Table Primers used for deep amplicon sequencing assay. (DOCX)

S2 Table Primers used for Sanger sequencing of individual worms. (DOCX)
