# Supplementary material for: Deep amplicon sequencing highlights low intra-host genetic variability of Echinococcus multilocularis and high prevalence of the European-type haplotypes in coyotes and red foxes in Alberta, Canada
Source: PLoS Negl Trop Dis. 2021 May 26;15(5):e0009428. doi: 10.1371/journal.pntd.0009428 (PMC8153462; doi:10.1371/journal.pntd.0009428)
Supplement: S1 Appendix — We aligned previously reported sequences found in North America of cox1, nad1 and cob genes to identify highly polymorphic regions and select the primers to amplify the target regions. The primers chosen per locus are shown underlined. The arrows indicate the 5’ to the 3’ direction. (DOCX) [file pntd.0009428.s001.docx]

**S1 Appendix. Multiple sequence alignment of *cob*, *cox1* and *nad1* genes**

We aligned previously reported sequences found in North America of *cox1*, *nad1* and *cob* genes to identify highly polymorphic regions and select the primers to amplify the target regions. The primers chosen per locus are shown underlined. The arrows indicate the 5' to the 3' direction.

**A**. *cob*

NC_000928_*wgs* 3050 GTTA**GTTTAAACTGGTAGATTGTGGTTC**TATTGAGTACTGTTTAGTTGTATTAGTAATGG 3110

NC_000928_*cob* 1 ATGATTGTTTTGTTTCGACGTAATTTAATAGATTTACCAATTAATTATTCTTTGAATTAT 60

SK1 ............................................................

BC1 ............................................................

E4 ............................................................

EAB ............................................................

ESK ............................................................

ECA ............................................................

N1 ............................................................

N2 ............................................................

*Eg* G10 ........G........G........G........................C.T......

*Eg* G8 ........G........G........G........................C.T..C...

NC_000928_*cob* 61 TATTGAAGTAGTGGGTTTGTATTGTCTATGTTTATGATTCTTCAAATTTTTACTGGAGTA 120

SK1 ............................................................

BC1 ............................................................

E4 ....................G.......................................

EAB ....................G.......................................

ESK ....................G.......................................

ECA ....................G.......................................

N1 ...........C................................................

N2 ...........C................................................

*Eg*_G10 ........C.....C........A........C...G.......G...........G..G

*Eg*_G8 .....G..C.....C.....G..A........C...........G...........G..G

NC_000928_*cob* 121 TTGTTGTCTTTTTTGTATGTAGCTGATTTTATGTGTAGATTTTTTATGGTTATGAATTTA 180

SK1 ............................................C...............

BC1 ............................................C...............

E4 ............................................C...............

EAB ............................................C...............

ESK ............................................C...............

ECA ............................................C...............

N1 ....................G.......................................

N2 ............................................................

Eg_G10 C...................G......................................G

Eg_G8 C....A..............G......................................G

NC_000928_*cob* 181 TCTAATGATTCTTTTTTTACTTGATGTTTGCGTTATTGGCATATGGTAGGTGTAAATGTA 240

SK1 ............................................................

BC1 ............................................................

E4 ............................................................

EAB ......................................................G.....

ESK ......................................................G.....

ECA ......................................................G.....

N1 ............................................................

N2 ............................................................

Eg_G10 ..........................C.....G.....A......A.T.....T.....C

Eg_G8 ..........................C.....G.....A......A.T.....T.....C

NC_000928_*cob* 241 CTGTTTATTTTGTTATTCTTTCATATGGGTATGGCTTTGTATTATGGTAGTTATGTTAAG 300

SK1 ............................................................

BC1 ............................................................

E4 ............................................................

EAB ............................................................

ESK ............................................................

ECA ............................................................

N1 ............................................................

N2 ............................................................

*Eg*_G10 T.............G..T..........................C.....C.........

*Eg*_G8 T.............G..T................................C.........

NC_000928_*cob* 301 AAGGGTGTTTGGAATGTTGGTTTTGTGTTATATTTGTTAGTTATGGGTGAGGCATTTACT 360

SK1 ............................................................

BC1 ............................................................

E4 ............................................................

EAB ............................................................

ESK ............................................................

ECA ............................................................

N1 ...............................G............................

N2 ............................................................

*Eg*_G10 ..........................A..G.....A..............A..G......

*Eg*_G8 ...........................C.G.....A..G...........A..G......

NC_000928_*cob* 361 GGTTATATTTTACCTTGGCGTCAGATG**TCTTATTGGGCTGCCACTGT**CCTTACTTCAATT 420

SK1 ........C..........A........................................

BC1 ........C..........A........................................

E4 ........C..........A........................................

EAB ........C..........A........................................

ESK ........C..........A........................................

ECA ........C..........A........................................

N1 ...................A...........................T............

N2 ...................A...........................T............

*Eg*_G10 .................A.A...A.................T..................

*Eg*_G8 .................A.A...A.................T..................

**B**. ***cox1_A***

NC_000928 421 AGTAGTGGTGTTGATTTTTTGATGTTTTCTTT**GCATTTAGCAGGTGTTTCTAGAG**TTTTT 480

ECA ............................................................

E4 ............................................................

EAB ............................................................

ESK ............................................................

N2 ............................................................

N1 ............................................................

BC1 .....G..............................................A.......

SK1 .....G......................................................

*Eg*_G10 ...TA.................................G.....................

*Eg*_G8 ...TA......A..........................G......T..............

NC_000928 481 AGTTCTATAAATTTTATTTGTACTTTGTATAGTGTTTTTATGACTAATGTATTTTCTCGG 540

ECA ............................................................

E4 ............................................................

EAB ............................................................

ESK ............................................................

N2 ............................................................

N1 ............................................................

BC1 ............................................................

SK1 ....................A...........G...........................

*Eg*_G10 ........C..A......A.............G..............A..G........T

*Eg*_G8 ........T..A......A.............G..........T...A..G........T

NC_000928 541 ACTTCTATTGTTCTTTGGTCATATTTATTTACTTCTATTTTATTGTTAGTGACGTTGCCT 600

ECA ............................................................

E4 ............................................................

EAB ............................................................

ESK ......G.....................................................

N2 ............................................A...............

N1 ............................................................

BC1 ............................................................

SK1 ......G.....................................................

*Eg_*G10 ........AA..........T.....G..............G.....G............

*Eg*_G8 ........AA.......A..T.....G...........C..G.....G............

NC_000928 601 GTTTTGGCTGCTGCTATTACTATGCTTTTGTTTGATCGTAAATTTTGTTCTGCTTTTTTT 660

ECA ............................................................

E4 ............................................................

EAB ............................................................

ESK ............................................................

N2 ............................................................

N1 ............................................................

BC1 ............................................................

SK1 ......................................A.....................

*Eg*_G10 .....A.......................A...........T..................

*Eg*_G8 .....A.......................A...........T..................

NC_000928 661 GATCCGTTAGGTGGTGGTGATCCTATTCTATTTCAGCATATGTTTTGGTTTTTTGGTCAT 720

ECA ..............G............T................................

E4 ..............G............T................................

EAB ..............G............T................................

ESK ..............G............T................................

N2 ...........................T................................

N1 ...........................T................................

BC1 ..............G............T................................

SK1 ..............G............T................................

*Eg*_G10 .....A..G..................T.......A........C...............

*Eg*_G8 .....A..G..................T.......A....................C...

NC_000928 721 CCGGAGGTTTATGTTTTGATTCTGCCTGGATTTGGTATAATTAGTCATATTTGTTTAAGT 780

ECA ............................................................

E4 ............................................................

EAB ............................................................

ESK ............................................................

N2 ..............G........................G....................

N1 ..............G.............................................

BC1 ............................................................

SK1 ............................................................

*Eg_*G10 ..A...........G......T..............G.T.................G..G

*Eg*_G8 ..............G..A...T.......G......G.T.................G..G

NC_000928 781 ATAAGTGGTAATTTTGATGCGTTTGGGTTTTATGGTTTGTTGTTTGCTATGTTTTCTATA 840

ECA .........................................A..................

E4 .........................................A..................

EAB .........................................A..................

ESK .........................................A..................

N2 ...................T........................................

N1 ...................T........................................

BC1 .........................................A..................

SK1 .........................................A..................

*Eg*_G10 ..T...TC......G....TT..............G........................

*Eg*_G8 ..T....C......G....TT..............G........................

NC_000928 841 GTGTGTTTAGGGAGTAGTGTTTGGGGTCATCATATGTTTA**CTGTTGGGTTGGATGTGAAG** 900

ECA ............................................................

E4 ............................................................

EAB ............................................................

ESK ............................................................

N2 ............................................................

N1 ............................................................

BC1 ............................................................

SK1 ............................................................

*Eg*_G10 ...........T..............A.......................A.........

*Eg*_G8 ...........T..............G....................A............

**C.** ***cox1_B***

NC_000928 841 GTGTGTTTAGGGAGTAGTGTTTGGGGTCATCATATGTTTA**CTGTTGGGTTGGATGTGAAG** 900

ECA ............................................................

E4 ............................................................

EAB ............................................................

ESK ............................................................

N2 ............................................................

N1 ............................................................

BC1 ............................................................

SK1 ............................................................

*Eg*_G10 ...........T..............A.......................A.........

*Eg*_G8 ...........T..............G....................A............

NC_000928 901 ACGGCGGTTTTTTTTAGTTCTGTTACGATGATTATAGGTGTTCCGACTGGTATAAAGGTG 960

ECA ............................................................

E4 ............................................................

EAB ............................................................

ESK ............................................................

N2 ............................................................

N1 ............................................................

BC1 ............................................................

SK1 ............................................................

*Eg*_G10 ..T..T....................T.................T...............

*Eg*_G8 ..T..T....................T..............C..T.....A.........

NC_000928 961 TTTACTTGGTTGTATATGTTGCTTAATTCTAGTGTAAATAAGAGTGATCCTATTTTGTGG 1020

ECA ............................................................

E4 ............................................................

EAB ............................................................

ESK ............................................................

N2 ............................................................

N1 ............................................................

BC1 .....................T......................................

SK1 ............................................................

*Eg*_G10 ....................AT.G.......A...T...TCT.........G........

*Eg*_G8 .....C........C......T.A.......A.......GGTG........G........

NC_000928 1021 TGGGTTATTTCTTTTATAGTGTTGTTTACGTTTGGTGGTGTTACTGGTATAGTTTTATCT 1080

ECA ............................................................

E4 ............................................................

EAB ............................................................

ESK ............................................................

N2 ............................................................

N1 ............................................................

BC1 ............................................................

SK1 ............................................................

*Eg*_G10 ....................T..A...........G..C.................G...

*Eg*_G8 ....................T..A..............C.................G...

NC_000928 1081 GCTTGTGTGTTGGATAATGTTTTACACGATACTTGATTTGTGGTGGCTCATTTTCATTAT 1140

ECA ............................................................

E4 ............................................................

EAB .........................................A..................

ESK ............................................................

N2 ............................................................

N1 ............................................................

BC1 ............................................................

SK1 ............................................................

*Eg*_G10 ..........................T.................................

*Eg*_G8 .........................................A..................

NC_000928 1141 GTTATGTCGTTAGGTTCTTATATAAGGATTGTTGTTATGTTTATTTGATGGTGACCGTTG 1200

ECA ............................................................

E4 ............................................................

EAB ............................................................

ESK ............................................................

N2 ............................................................

N1 ............................................................

BC1 ............................................................

SK1 ............................................................

*Eg*_G10 .......................G..T...A................G..A..G..T...

*Eg*_G8 .......................G..T...A................G.....G..T...

NC_000928 1201 ATTACTGGTTTGAGGTTGAATAAGTGTTTGTTACAATGTCAGTGTATAATTTCTAATATT 1260

ECA ............................................................

E4 ............................................................

EAB ............................................................

ESK ............................................................

N2 ............................................................

N1 ............................................................

BC1 ............................................................

SK1 ............................................................

*Eg*_G10 ........C.....A...................................C........A

*Eg*_G8 ........C.....A.................G........A.................A

NC_000928 1261 GGGTTTAATCTTTGTTTTTTTCCTATGCATTATTTTGGTTTATGTGGGT**TGCCTCGTCGT**  1320

ECA ............................................................

E4 ............................................................

EAB ............................................................

ESK ............................................................

N2 .....................................................G......

N1 ................................C....................G......

BC1 ............................................................

SK1 ............................................................

*Eg*_G10 ..T.................C....................G...........A......

*Eg*_G8 ..T................................C.....G...........A......

NC_000928 1321 **GTGTGTATA**TATGAGTGTAGATATAATTGGGTTAAAATGGTTTGTACTGTTGGTTCTTTT 1380

ECA ............................................................ 1380

E4 ............................................................ 1380

EAB ............................................................ 1380

ESK ............................................................ 1380

N2 ..............................A............................. 1380

N1 ............................................................ 1380

BC1 ............................................................ 1380

SK1 ............................................................ 1380

*Eg*_G10 ........C.....A.A...C.........A....TG..........G............ 1380

*Eg*_G8 ........T.....ACA...G.........A....TG..........G............ 1380

**D. *nad1***

NC_000928 21 AATAAGGTTGGTTTTGCTGGTTTGTTGCAGAGATT**TGCTGATTTGTTAAAGTTAGTGATC** 180

*Eg*_G10 ............A.CAT.......C.......G..............G........A..T

BC1 ........................................................

M1 .............................................

M2 .............................................

Gesy_E .............

Gesy_F .............

Gesy_A .............

NC_000928 181 AAGTTTAAGAATTTTTATTTTCAAAGTCGTAGGTATATTGGTTTGTTGGGCGTTTTTTTG 240

*Eg_*G10 ....................C..G............G.......A..T........G...

BC1 ..................................................T.........

M1 ............................................................

M2 ............................................................

Gesy_E ..................................................T.........

Gesy_F ..................................................T.........

Gesy_A ..................................................T.........

NC_000928 241 TTAATAATTTTGGTTATTATATATTCTTTTATTTATGGTAGATATTATAGTGTTAGTTAT 300

*Eg*_G10 ..G...G........G.GG.G.....G.......................A.........

BC1 ............................................................

M1 ............................................................

M2 ............................................................

Gesy_E ............................................................

Gesy_F ............................................................

Gesy_A ............................................................

NC_000928 301 AATAGTCTTTCAGTATTGTGGTTTTTAGCTGTTGCTAGTATTTCTAGGTATTCTTTGTTG 360

*Eg*_G10 .G...G.....T.....A..A..........C.T....A.....................

BC1 ............................................................

M1 ............................................................

M2 ............................................................

Gesy_E ............................................................

Gesy_F ............................................................

Gesy_A ............................................................

NC_000928 361 TGTGCTGGTTGGGGTAGTTACAATAAATATTCGTTTTTAAGTTCTGTTCGATGTGCTTTT 420

*Eg_*G10 .................C..T....GT.....T........G..G...............

BC1 ............................................................

M1 ............................................................

M2 ....T.......................................................

Gesy_E ............................................................

Gesy_F ............................................................

Gesy_A ....T.......................................................

NC_000928 421 GGGTCTGTTAGGTTTGAAGCTTGTTTTATGTGTGTGGTAATTTTTTGTTCTTTGTGTTAC 480

*Eg*_G10 ..A...................................T........CG....A....GT

BC1 ............................................................

M1 ............................................................

M2 ............................................................

Gesy_E ............................................................

Gesy_F .........................................G..................

Gesy_A ............................................................

NC_000928 481 TGTAGGTATAATTTGATTGATTTTTATTATAGTTGTTGATGAAG**CTTGTTGTTGTTTCCA** 540

*Eg*_G10 ...G..........A............C......A...G..T..-.....A..A..C...

BC1 ............................................................

M1 ............................................................

M2 ...............G............................................

Gesy_E .........................................................

Gesy_F .........................................................

Gesy_A .........G...............................................

NC_000928 541 **TTGATTTATG**GATTGTTTTTGGTGTGTGTGCTATGTGAGACTAATCGTATACCATTTGAT 600

*Eg*_G10 ..A........G..A..C............T.G................CT.........

BC1 ............................................................

M1 .................................................CT.........

M2 .................................................CT.........
