# Supplementary material for: Deep amplicon sequencing highlights low intra-host genetic variability of Echinococcus multilocularis and high prevalence of the European-type haplotypes in coyotes and red foxes in Alberta, Canada
Source: PLoS Negl Trop Dis. 2021 May 26;15(5):e0009428. doi: 10.1371/journal.pntd.0009428 (PMC8153462; doi:10.1371/journal.pntd.0009428)
Supplement: S1 Table — (DOCX) [file pntd.0009428.s002.docx]

**S1 Table.** **Primers used for deep amplicon sequencing assay**.

| **Target region** | **Primer Name** | **Oligonucleotide sequence (5’–3')** | **Annealing** | **Reference** |
| --- | --- | --- | --- | --- |
| **Amplicon size** | **Direction F/R** |  | **temp. (°C)** |  |
| *cob*: 1-387  431 bp | Cob_N - F | Illumina adapter sequence – 0-3N’s – GTTTAAACTGGTAGATTGTGGTTC | 63 | [11] |
|  | Cob_388 - R | Illumina adapter sequence – 0-3N’s – ACAGTGGCAGCCCAATAAGA |  | This study |
| *cox1_A*: 476-880  448 bp | Cox1_A - F | Illumina adapter sequence – 0-3N’s – GCATTTAGCAGGTGTTTCTAGAG | 64 | This study |
|  | Cox1_A - R | Illumina adapter sequence – 0-3N’s – TCTTCACATCCAACCCAACAG |  |  |
| *cox1_B*: 901-1309  448 bp | Cox1_B - F | Illumina adapter sequence – 0-3N’s – CTGTTGGGTTGGATGTGAAG | 64 | This study |
|  | Cox1_B - R | Illumina adapter sequence – 0-3N's – TATACACACACGACGAGGCA |  |  |
| *nad1*: 180-524  395 bp | Cest1 - F | Illumina adapter sequence – 0-3N’s – TGCTGATTTGTTAAAGTTAGTGATC | 62 | [27] |
|  | Cest2 - R | Illumina adapter sequence – 0-3N’s – CATAAATCAATGGAAACAACAACAAG |  |  |
