# Supplementary material for: Deep amplicon sequencing highlights low intra-host genetic variability of Echinococcus multilocularis and high prevalence of the European-type haplotypes in coyotes and red foxes in Alberta, Canada
Source: PLoS Negl Trop Dis. 2021 May 26;15(5):e0009428. doi: 10.1371/journal.pntd.0009428 (PMC8153462; doi:10.1371/journal.pntd.0009428)
Supplement: S2 Table — (DOCX) [file pntd.0009428.s003.docx]

**S2 Table. Primers used for Sanger sequencing of individual worms.**

| **Target** | **Oligonucleotide sequence (5'–3')** | **Amplicon size (bp)** |
| --- | --- | --- |
| **Direction F/R** |  |  |
| *cob -* F | GTTTAAACTGGTAGATTGTGGTTC | 1068 |
| *cob -* R | CTCCACAGTAGAAATCACCATCA |  |
| *nad2 -* F | GCGTTGATTCATTGATACATTGT | 882 |
| *nad2 -* R | TAGTAAAGCTCAAACCGAGTTCT |  |
| *cox1-* F | GACTTTCTCTTTGGTTGGTGTAAG | 1608 |
| *cox1-* R | AACCTAAACAACCAACTTCACAG |  |

Reference: Nakao, et al. [11]
